# Foreign Correspondence

**Published:** 1843-04-29

**Authors:** 


### FOREIGN CORRESPONDENCE.

*Diminished number of Medical Students in Paris—Dr. Quadri, the Neapolitan Surgeon—Singular case of the influence of the imagination on the fœtus—Dr. Leroy d'Etiolles—Electro puncture—Professor Velpeau—Dr. Briquet and Large Doses of Quinine—Dr. Louis—The appointment of Dr. Baudens to Val-de-Grace—The discussion on Tenotomy—The vacant seat at the Institute—Dr. Andral—M. Dumas—Dr. Orfila and the Case of Poisoning by Prussic Acid—M. Renault—Climate and health of the Metropolis during the Winter.*

Paris, February 21, 1843.

To the Editor of the Medical Examiner.

Sir—The number of medical students attending the different schools in France has been gradually decreasing since the year 1835—the period when new regulations were enforced, and greater requirements exacted of the aspirants for degrees. By an official return lately made to the Minister of Public Instruction, it is shown that the number of new students, inscribed for the present scholastic year, is nearly two-thirds less than in 1835. The numbers being, of new Inscriptions,

| | |
|---|---|
| For the year 1835–'36, | 1,522. |
| " " 1842–'43, | 628. |

This falling off is particularly felt at Paris, where there are only 200 new matriculants for the present term; whereas the number has heretofore been from 500 to 700—(in 1835, 900.) A new organization of the Primary and Secondary Medical Schools of the Provinces, by rendering them efficient, has had the effect of keeping young men in the vicinity of their homes, who have hitherto been compelled to come to the metropolis for advantages which were then nowhere else afforded them. The Professors of the Paris faculty complain loudly of the thinness of their classes; for, although being paid by the government, they experience no pecuniary loss, they are dissatisfied at seeing their benches, usually so crowded, now comparatively empty; indeed, with the exception of one or two of the *Cliniques*, and during the lectures of Monsieur Orfila, the rooms may be said to be abandoned.

This decrease in the number of candidates for medical degrees may be considered a fortunate circumstance, for France is abundantly supplied with physicians, and the profession as much overstocked as in our own country. The proportion of practitioners in Paris, including the *Officiers de Santé*, is nearly one for every 600 inhabitants; and in the Provinces, about one to 900.

Although the number of native students is thus decreasing, there are at present an unusual number of foreign ones attending the private lectures and walking the hospitals, and amongst them many intelligent and attentive Americans.

Monsieur Quadri, the distinguished Italian surgeon, of Naples, has lately been on a visit to Paris, where he has met with a warm reception from his professional brethren, who have vied with each other in their attentions to him. He is a man of mild and quiet manner, agreeable conversation, and a prepossessing appearance. Having been for a number of years engaged in the study of diseases of the eye, and become one of the highest authorities on this subject, he was requested to read to the Royal Academy of Medicine a memoir on the "Ophthalmiæ of

Italy," which was listened to with the greatest attention by the learned body to which it was addressed. After an absence of a few weeks, M. Quadri has now returned to resume his professional duties at Naples.

We have lately had here, in the person of a new-born infant, another powerful proof of the influence of the mother's imagination in the production of phenomena which are supposed to occur during the earlier periods of pregnancy. The infant, which was born a few weeks since, was found at its birth to have the body covered with large spots, or patches, shining, and of a brownish black color, presenting the appearance of smoked or burned meat. The mother, when questioned by her medical attendant, recollected that, at the period of the dreadful railway accident, which happened on the 8th of last May, on the Paris and Versailles railroad, she accompanied her husband to see the remains of the unfortunate victims who had been burned to death; the sight was a shocking one, and made a most disagreeable impression upon her, which lasted for several days, and even nights,—for she says that she frequently dreamed of the dreadful spectacle. The lady was at that period in the second month of her pregnancy; and such members of the faculty as I have conversed with here on the subject, agree in attributing the phenomenon to the impression made on the mother's mind in the manner and at the period indicated. The child, with the exception of these marks, is like all children of its age, and perfectly well-formed and healthy.

Dr. Leroy d'Etiolles has been engaged lately in some interesting experiments at the Hôtel Dieu, with the view of testing the influence of *Electro-puncture* in promoting absorption. I have assisted him in several operations, which have so far, however, been confined to hydrocele: the cases selected were of long standing, but in other respects favourable. A needle was introduced into the cavity of the tunica vaginalis, and another into the subcutaneous cellular tissue of the scrotum; these were then connected with the poles of a small galvanic battery, and the acid solution poured on to the plates. The patients complained, at first, of a shock, and afterwards of a disagreeable and slightly painful *burning* sensation. The galvanic action was continued for fifteen or twenty minutes on each occasion, and renewed about every sixth day. In two cases that I have watched closely, the cure was completed after the third application of electricity; i. e., the whole of the effused liquid had been absorbed; it remains, however, to be seen whether the disease will not recur. This method has not yet been reduced into a regular system of treatment, and until some time shall have elapsed, and a greater number of experiments been made, it will be impossible to form a just estimate of its value as a therapeutic means, in cases of serous effusion.

Professor Velpeau has likewise been engaged in experiments, which, if not more important, are certainly more daring than those of Dr. Leroy. I allude to his injection of a solution of iodine into the joints, in cases of hydrarthrosis. I have seen some of the patients, treated by him in this way, and they were certainly benefitted, if not cured by it. As the Professor is about addressing a communication to the Academy of Sciences on the subject, I shall await its publication before entering into the details of his cases. I may remark, however, that in a recent clinical lecture he stated his intention of generalizing the method, and applying it to the first favourable cases of ascites and hydrothorax that should present themselves!!!

The large doses of quinine administered a few years back in our southern country in cases of yellow fever, without producing serious consequences, seems to have engendered amongst the French physicians a desire to test its influence in other diseases, when given in the same way.

Dr. Briquet, one of the physicians to the Hôpital Cochin, has tried its effect in the various forms of rheumatism, and lately published an account of eighteen cases of acute articular rheumatism which had been completely cured, and in a short time, by very large doses of the sulphate of quinia, continued during six or eight consecutive days. These cases being well authenticated, other physicians adopted the practice, and were generally successful until within the last few weeks, when the supervention of some fatal accidents has, for the moment, damped their zeal in favour of Dr. Briquet's method. Not less than five persons are reported as having died from the effects produced by the ingestion of such large quantities of the medicine as were given—two of them in the wards of the gentleman with whom the practice originated.

The fact that quinine, when properly administered, and in reasonably large doses, is capable of relieving both acute and chronic articular rheumatism, is now well established. I have myself seen some cases successfully treated by it, and amongst others, a remarkable one, to which my attention was directed by Dr. Louis, at the Hôpital Beaujon. It was a female patient, who suffered intensely when admitted into the hospital, and who was entirely and completely cured after taking *four grammes* (72 grains) of quinine *per diem*, during eight consecutive days. Louis is of opinion that the error heretofore has consisted in administering the medicine in too concentrated a form. He has been in the habit of dividing the doses, and giving them in a large quantity of vehicule, and, as yet, has had no unfortunate consequences to ensue.

Some little dissatisfaction has been created amongst the medical corps of the army, by the recent promotion of Dr. Baudens, who is comparatively a young man, to be First Professor and Chief Surgeon to the Military School and Hospital of Val-de-Grace. Although a meritorious officer, and accomplished surgeon, the preference given to Dr. B. over so many of his seniors, is attributed to the influence of an important member of the Royal family, to whom he is professionally attached.

The long pending discussion at the Royal Academy of Medicine, on the subject of tenotomy, after occupying some eight or ten meetings, has at last been brought to a conclusion, without, however, much new light having been thrown on the subject; indeed, this has been a personal matter throughout, and conducted in so objectionable a manner as to derogate from the dignity of the Academy, and rather detract from, than add to, the reputation of those engaged in it.

Considerable excitement is occasioned in the Medical Society here, as the period approaches for the elections to supply the places of Larrey and Double in the Royal Institute. It appears to be generally thought that Lallemand, of Montpellier, or Velpeau will be chosen for the surgical section; and Andral or Cruveilhier for that of medicine. The two former gentlemen will, however, have to contend against Civiale, who is supported by the powerful influence of Arago. And it is probable that neither

Andral nor Cruveilhier would be selected, if Louis would consent to become a candidate.*

Monsieur Dumas, the chemist, has just been elected a member of the Royal Academy of Medicine.

The *Gazette des Tribunaux* contains, in one of its late numbers, the account of an interesting medicolegal case which has just been decided at Chambèry. A man of the name of Héritier was accused of having poisoned his uncle with prussic acid. The physicians who had attended the uncle during his last moments were called as witnesses, and also directed to assist at the autopsy which the authorities had ordered to be made. These gentlemen, as well as the chemists employed to analyse the contents of the stomach and the liquids of the body, testified; the former, that their patient had died with all the symptoms of poisoning by cyanhydric acid; and the latter, that they had detected the presence of the same poison in such portions of the body of the defunct as had been submitted to their examination. This, and other evidence was so strong against the prisoner, that the jury were about bringing in a verdict of guilty, when the counsel for Héritier succeeded in obtaining a suspension of the proceedings until he could consult some of the higher medico-legal authorities. Dr. Orfila, the Dean of the Medical Faculty of Paris, and Professor of Chemistry, was applied to, and having examined closely all the circumstances of the case, succeeded in demonstrating, most clearly and satisfactorily,

1st. That the symptoms which preceded the death of the person supposed to have been poisoned, were not such as to justify the assertion that he had died from poisoning.

2d. That the chemists employed were mistaken in thinking that they had detected Prussic acid in the parts examined by them; and

3d. That there was every reason for supposing that the uncle had died of an attack of apoplexy, to which he had been subject during life; the symptoms in the case being such as might be expected in apoplexy; and the fact of the existence of a large clot of blood in one of the ventricles of the brain, tending to confirm such a diagnosis.

The paper of Dr. Orfila, when read by the prisoner's counsel, produced an immense sensation in court, and was sufficient of itself to outweigh all other testimony, as was proved in the sequel, by the full acquittal of the prisoner Héritier.

At one of the late meetings of the Royal Academy of Medicine, Monsieur Renault presented some pathological pieces from a horse, which had died eight days after having had injected into his jugular vein some blood which had been drawn from the jugular vein of another horse labouring under glanders. The pieces presented exhibited the lesions characteristic of acute glanders, and the experiment of M. Renault was considered as conclusive, with regard to the capability of communicating this dreadful malady by transfusion of the diseased blood. The same morbid blood that was injected into the veins of the healthy horse, when examined under a powerful microscope, was not found to differ in any respect from that liquid when in a perfectly normal state.

The winter has been uncommonly mild for Paris; and it is to this circumstance that is attributed the great prevalence of spring and early summer complaints, some of which, such as measles and scarlet fever, have lately become epidemic. Varioloid is also very prevalent; but notwithstanding this, the mortality of the Capital does not appear to be greater than is usual at this season of the year; and all diseases now prevailing assume rather a mild form. Within the last few days a change has occurred in the temperature, and we have rain and snow, with the thermometer at 30° to 35° of Fahrenheit.

F. C. S.

*Since the above was written the election to the medical section has been made, and Dr. Andral is the individual chosen. Four candidates were proposed by the "Election Committee." These were Andral, Guérin, Cruveilhier and Poisseuille. The vote stood, for

| | |
|---|---|
| Andral, | 42 |
| Cruveilhier, | 4 |
| Poisseuille, | 4 |
| J. Guérin, | 5 |

